# Use of Bland-Altman Analysis to Examine the Racial and Ethnic Representativeness of Study Populations in Community-Based Pediatric Health Research

**DOI:** 10.1001/jamanetworkopen.2023.12920

**Published:** 2023-05-11

**Authors:** Danielle M. Krobath, Elena N. Naumova, Adolfo G. Cuevas, Jennifer M. Sacheck, Norbert L. W. Wilson, Christina D. Economos

**Affiliations:** 1 Friedman School of Nutrition Science and Policy, Tufts University, Boston, Massachusetts; 2 Eliot-Pearson Department of Child Study and Human Development, Tufts University, Medford, Massachusetts; 3 Department of Community Health, Tufts University, Medford, Massachusetts; 4 Department of Social and Behavioral Sciences, School of Global Public Health, New York University, New York, New York; 5 Center for Anti-racism, Social Justice, and Public Health, New York University, New York, New York; 6 Milken Institute School of Public Health, The George Washington University, Washington, DC; 7 Duke Divinity School, Duke University, Durham, North Carolina; 8 Sanford School of Public Policy, Duke University, Durham, North Carolina

## Abstract

**Importance:**

Children from marginalized racial and ethnic groups are underrepresented in health research. To improve external validity and routinize race and ethnicity reporting, a specific and standardized methodology for quantifying representativeness of participant populations is needed.

**Objective:**

To develop a standardized method for quantifying the racial and ethnic representativeness of study samples.

**Design, Setting, and Participants:**

In this cross-sectional study, data from 7 US community-based health studies (conducted between 2003 and 2017) were retrospectively pooled to assess the school-level representativeness of enrolled samples by race and ethnicity. The sampling frame for the study was constructed using the National Center of Education Statistics Common Core of Data, which provides year-specific racial and ethnic counts by grade. Representativeness was quantified by aggregating children’s data at the school level, reported individually for Asian, Black, Hispanic or Latino, Native Hawaiian or other Pacific Islander, White, or multiple races. In this analysis, the Asian and Native Hawaiian or other Pacific Islander subgroups were combined. Data were analyzed from April 1 to June 15, 2022.

**Exposure:**

Community-based nutritional health studies conducted with children in grades 1 to 8.

**Main Outcomes and Measures:**

Visual comparisons of percentage expected and percentage observed of the pooled sample by race and ethnicity were performed using scatterplots and Bland-Altman plots. Spearman rank-order correlation was used to assess associations.

**Results:**

This study included 104 study schools (N = 5807 children) located in California, Kentucky, Massachusetts, Mississippi, and South Carolina. Bland-Altman analysis revealed notable patterns and variability in the representativeness of racial and ethnic groups. Differences in the overall representativeness of Asian or Native Hawaiian or other Pacific Islander children (0.45 percentage points [95% CI, −7.76 to 8.66]), Black children (0.12 percentage points [95% CI, −15.73 to 15.96]), and White children (−0.72 percentage points [95% CI, −23.60 to 22.16]) were negligible, but measures of spread suggested that target population demographics affected representativeness differently across groups.

**Conclusions and Relevance:**

The results of this cross-sectional study suggest that replicating, testing, and scaling the proposed method for quantifying racial and ethnic representativeness, which uses measures of spread, could improve the transparency of race and ethnicity reporting during publication and lead to a more externally valid health evidence base. During implementation, investigators should adopt community-based research methods and allocate appropriate resources during recruitment, including a priori assessment of population demographics, as these conditions may affect racial and ethnic study enrollment differently. Prioritizing these methodological decisions could alleviate rising inequities.

## Key Points

**Question:**

How can reporting of racial and ethnic representativeness in pediatric research studies be optimized for specificity and replication to address methodological gaps and reduce health disparities?

**Findings:**

In this cross-sectional study with a pooled sample of 104 schools located in 5 US states, the Bland-Altman method was adapted to quantify school-level racial and ethnic representativeness using measures of spread. Overall representativeness was observed, yet group means obscured the substantial within-group disparities observed with residuals on Bland-Altman plots.

**Meaning:**

These results suggest that replication of the proposed Bland-Altman method may improve race and ethnicity reporting, representative recruitment, and external validity of the health evidence base.

## Teaser

This cross-sectional study examines the utility of Bland-Altman analysis in quantifying the racial and ethnic representativeness of community-based populations in pediatric health research.

## Introduction

Ensuring that marginalized racial and ethnic populations are well represented in research is essential to prevent growing health inequities. In the US, the health evidence base fails to reflect the true diversity of the population.^1,2^ Black, Indigenous, and Hispanic populations are systematically excluded from health research, regardless of their awareness of and willingness to participate in studies.^2,3,4^ Once enrolled, these groups often exhibit lower retention rates than their White peers.^1,3,5,6^ Disproportionate enrollment and retention compounds inequities^3,7^ disproportionately conferring any potential benefits of study participation.^8^ Additionally, misrepresentation impedes the external validity of the evidence base and extends to suboptimal health policies, programs, and health care services.^3^ Investigators could improve the representativeness of study populations by consistently reporting participant race and ethnicity and sample representativeness.^1,3,9,10,11^ However, the current paradigm for quantifying representativeness lacks consistency and specificity, ultimately hindering any advances in equity. At a minimum, addressing the causes of preventable racial and ethnic health disparities begins by ensuring that study samples reflect the demographics of the US.^7^

The longstanding consensus rightfully acknowledges the overrepresentation of White individuals in study populations and the underrepresentation of marginalized racial and ethnic groups. Yet specific representation patterns may be biased by the endogeneity of race and ethnicity reporting, since public disclosure of study demographics is not mandated by journals or funders; thus, investigators often forego this reporting. For example, Rees et al^12^ examined 612 pediatric clinical trials published in top medical journals between 2011 and 2020, and found that 27.7% did not report participant race or ethnicity. Based on studies that provided data on the race of participants, the authors concluded that Black participants were enrolled in a greater proportion than their share of the US population aged younger than 19 years and were therefore overrepresented.^12^ Flores et al^13^ assessed reporting of race and ethnicity in 230 US vaccine trials, and they found that all studies reported participant age and sex but only 58.3% reported race and only 34.3% reported ethnicity.^13^ These reporting discrepancies may affect our understanding of racial and ethnic study representativeness, as findings rely on investigator transparency.^14^

When study demographics are reported in peer-reviewed literature, representativeness is traditionally quantified by comparing the overall share of each racial and ethnic group enrolled in the study against their mean share of the population without accounting for age groups or locations.^1,7,12,15,16^ This approach could obscure disparities and provides little insight into recruitment procedures and the association between factors in the environment and enrollment within and across racial and ethnic groups. Overall, the health research community could benefit from a specific and consistent approach for assessing and reporting the racial and ethnic representativeness of study populations.

Here, we describe a methodology for quantifying the racial and ethnic representativeness of study populations and demonstrate its utility by compiling a convenience sample of data from 7 US community-based pediatric health studies. All studies, conducted by members of our team, collected parent- and guardian-reported child race and ethnicity and recruited at public schools. Investigators allocated extensive resources a priori to identify, partner with, and recruit from schools in communities serving predominantly marginalized populations to ensure that they were well represented (Table 1). Investigators aimed to enroll samples representative of each school’s diverse population. We hypothesized that there would be no differences in mean representativeness across racial and ethnic groups. Our overall objective is to contribute to a more equitable research paradigm by presenting a standardized methodology for quantifying the representativeness of study samples.

**Table 1.** Descriptions of Public Schools Nested Within Community-Based Pediatric Health Studies Included in Racial and Ethnic Representativeness Assessment^a^

Study	Year enrolled	State	Locale	No. of public schools (N = 104)	No. of participants included in analysis (N = 5807)	Grade	Study design	School or community recruitment criteria	Intervention or exposure	Comparison level
A	2016-2017	Massachusetts	Urban and suburban	18	882	3-4	Cluster RCT	Schools with >40% of student body eligible for FRPL or >40% non-White student body	School-level random assignment to intervention or control	Changes among children in intervention schools vs in control schools
B	2013	Massachusetts	Urban	8	418	3-6	Observational cross-sectional	Schools from a district with >50% Hispanic or Latino students and >50% receiving FRPL	No exposure	Descriptive data across all children
C	2011-2012	Massachusetts	Urban	12	632	4-8	Randomized double-blind clinical trial	Schools with a diverse racial and ethnic student body and >60% eligible for FRPL	Individual-level random assignment of children into 3 intervention groups	Changes in children based on group exposure level
D	2009-2010	Massachusetts	Urban	7	225	4-8	Observational cross-sectional	Schools from a district with high childhood overweight and obesity prevalence (49% had BMI >85th percentile)	No exposure	Descriptive data across all children
E	2008-2009	California, Kentucky, Mississippi, and South Carolina	Rural	8	1245	1-6	RCT	Rural school districts	Random assignment with 2 participating matched communities per state, which were each randomly assigned to intervention or control	Changes in children in intervention schools vs control schools
F	2007-2008	California, Kentucky, Mississippi, and South Carolina	Rural	22	919	1-6	Pre-to-post program evaluation	Rural school districts	All children received exposure	Within-participant changes
G	2003-2004	Massachusetts	Urban and suburban	29	1486	1-3	Nonrandomized community-level intervention with matched control communities	Intervention community selected based on preexisting partnership; control communities matched by city-level traits, including (1) non–English-speaking homes, (2) median household income, and (3) residents living below the FPL	Nonrandom assignment with 1 intervention community and 2 matched control communities	Changes in children in intervention schools vs control schools

Abbreviations: BMI, body mass index (calculated as weight in kilograms divided by height in meters squared); FPL, federal poverty line; FRPL, free or reduced-price lunch; RCT, randomized clinical trial.

^a^ Informed consent was received for 6325 children but race and/or ethnicity were not available for comparison for all children. eAppendix 1 and eTable 2 in Supplement 1 provide exclusion criteria and counts per study.

## Methods

The Tufts University Institutional Review Board exempted this cross-sectional study from review because it did not constitute human participant research. For the 7 pediatric research studies described herein, informed consent was obtained from the parents or caregivers of participants. The current study followed the Strengthening the Reporting of Observational Studies in Epidemiology (STROBE) reporting guideline.

### Bland-Altman Method

The Bland-Altman approach is widely accepted and applied in clinical settings to analyze the agreement between 2 quantitative measures.^17^ Bland and Altman^17^ developed a graphical technique with simple calculations for validation of measurement tools and techniques against preestablished clinical gold standards to overcome limitations of the alternative, based solely on correlation and regression analyses. The Bland-Altman method quantifies agreement between the 2 measures by assessing the mean differences of each observation and estimating the limits of agreement (ie, the range in which 95% of the differences between the first and second measures fall).^18^

Bland-Altman plots have been used previously in health disparities research. Wong et al^19^ compared racial and ethnic discrepancies in pulse oximetry and arterial oxygen saturation measures. In the current study, we visualized differences between the percentage expected for representativeness and the percentage enrolled for the following racial and ethnic subgroups: Asian, Black, Hispanic or Latino, Native Hawaiian or other Pacific Islander, White, and multiple races (eAppendix 1 in Supplement 1). We standardized our findings with percentage points to assess deviations from proportionate representation.

### Study Population and Data Source

To compute the share of each group expected for representativeness (herein referred to as *percentage expected*), we used the National Center for Education Statistics (NCES) Public Elementary/Secondary School Universe Survey.^20^ These data are compiled into the Common Core of Data, which provides the total race and ethnicity enrollment counts by sex and grade for all US public schools. It is an authoritative sampling frame for pediatric research and widely relied on by investigators and in population surveillance surveys.^21^ We calculated percentage expected by dividing the total number of enrolled students in each racial and ethnic group in the target grades by the number of all enrolled in those grades. By deriving percentage expected from the schools’ true and precise population parameters, it can be conceptualized as the gold standard for proportionate representativeness.^17,22^

To compute the share of each group that enrolled in studies (herein referred to as *percentage observed*), we pooled data from 7 community-based health studies led by members of our research team across 104 schools in 5 states, including California, Kentucky, Massachusetts, Mississippi, and South Carolina. Individual measures from children in grades 1 to 8 who enrolled in nutritional health studies were aggregated by the school where they were recruited (Table 1). Prior to baseline data collection, parents or guardians were asked to report child demographics, including grade, race, and ethnicity. Child race and ethnicity data were aggregated according to NCES reporting standards (eAppendix 1 in Supplement 1). In this analysis, the Asian and Native Hawaiian or other Pacific Islander subgroups were combined. We calculated percentage observed at the school level by dividing the number of enrolled children in each racial and ethnic group by the total number of study participants from each school.

We identified each study school in the NCES Common Core of Data and calculated school-level differences between percentage expected and percentage observed for each race and ethnicity. Additional details are provided in eAppendix 2 in Supplement 1.

### Interpretation of Bland-Altman Analysis to Reveal Representativeness Patterns

Key components of the Bland-Altman method reveal important information regarding site-level racial and ethnic representativeness. The process for quantifying representativeness is explained further in eTable 1 in Supplement 1. Scatterplots and Bland-Altman plots are presented in pairs, and comparisons were made separately for each racial and ethnic group. Points on graphs represent individual schools.

On the scatterplots, the x-axis presents the percentage expected and the y-axis is the percentage observed. The solid line is the line of identity, and the dashed line is the best fit. Perfect overlap of the line of identity and best fit indicates proportionate representation across schools.

On the Bland-Altman plots, the x-axis denotes the mean of percentage expected and percentage observed. The y-axis indicates percentage observed minus percentage expected. The shorter-dashed line shows the mean of the absolute value of the difference between the expected and observed percentages; its distance from the line of 0 on the y-axis (herein referred to as Y,0) is the overall percentage-point amount that the racial or ethnic group was represented across studies. The longer-dashed lines indicate the SDs (2) for this range; thus, wider intervals indicate greater variability in overall representativeness. Points along Y,0 are schools that were representative. Points above Y,0 are schools where each group was overrepresented, while schools below Y,0 were underrepresented. The distance between a point and Y,0 denotes the percentage-point distance from proportionate representation.

### Statistical Analysis

Schools were the primary unit of analyses. We visually inspected scatterplots and Bland-Altman plots to quantify representation patterns. To assess the association between percentage expected and percentage observed for each racial and ethnic group, we used Spearman rank-order correlation coefficients and a 2-sided α level of .01 to establish statistical significance. All analyses were conducted from April 1 to June 15, 2022, using Stata SE software, version 17 (StataCorp LLC).

## Results

**Figure 1.**
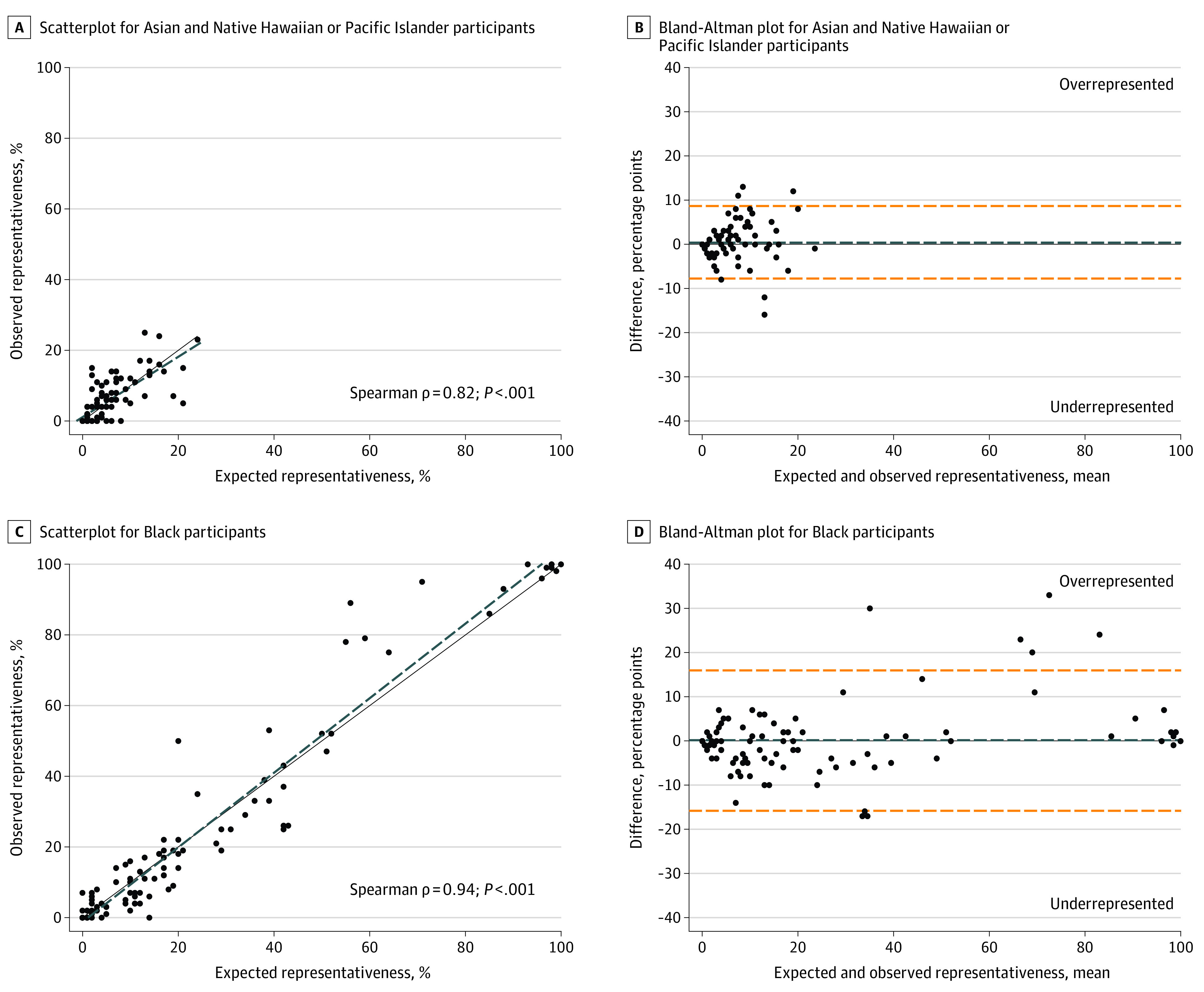
Differences Between Percentage Expected and Percentage Observed for Asian or Native Hawaiian or Other Pacific Islander Children and Black Children A and C, Scatterplots in which the dashed lines indicate the best-fit line and the solid lines indicate the line of identity. B and D, Bland-Altman plots in which the blue dashed lines indicate the mean of the absolute value of the difference (percentage expected minus percentage observed) and the orange dashed lines indicate the SDs (2) for this range. Points along Y,0 indicate schools that demonstrated perfect representativeness of the group. Points above Y,0 indicate schools where the group was overrepresented. Points below Y,0 indicate schools where the group was underrepresented.

**Figure 2.**
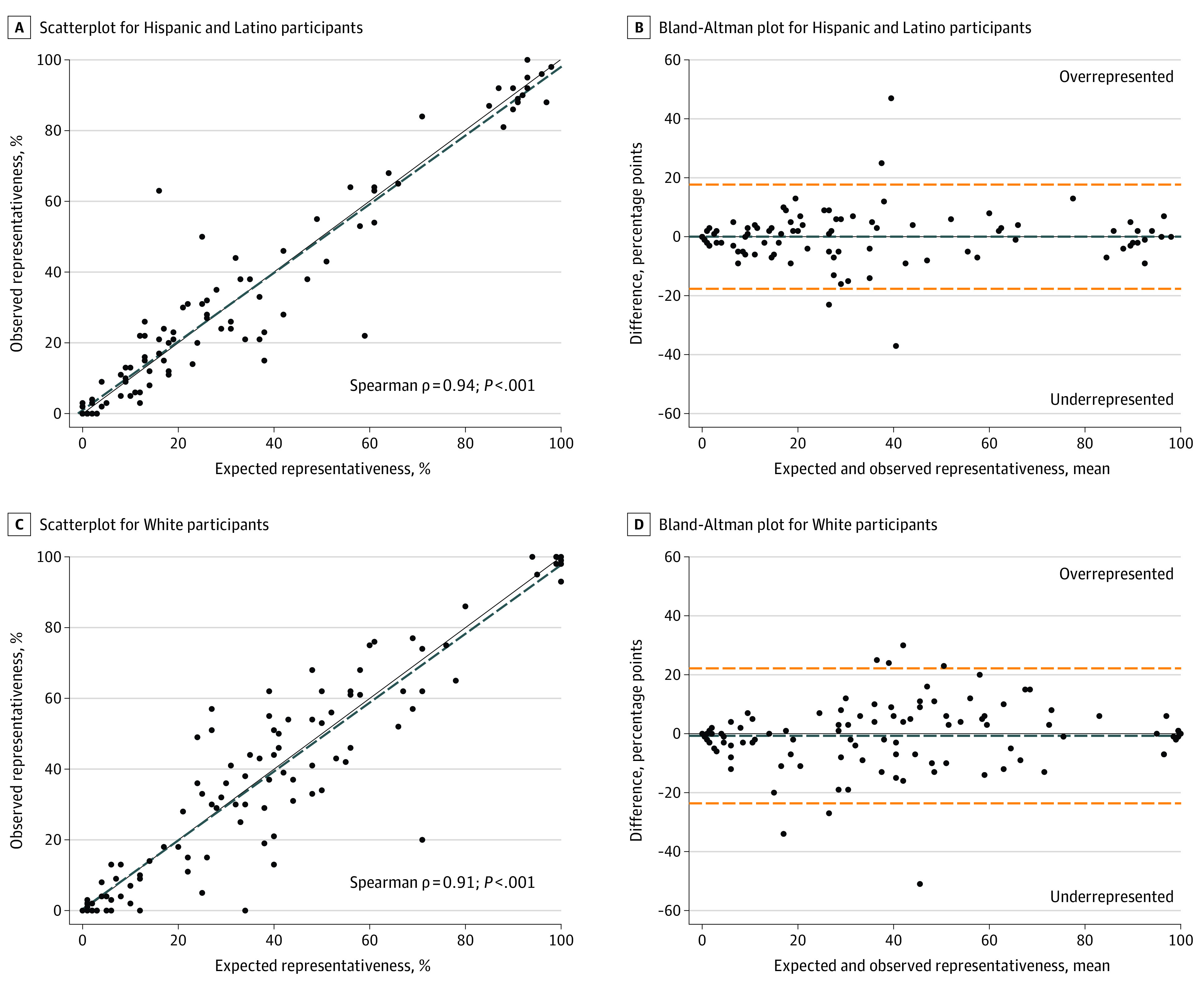
Differences Between Percentage Expected and Percentage Observed for Hispanic Children and White Children A and C, Scatterplots in which the dashed lines indicate the best-fit line and the solid lines indicate the line of identity. B and D, Bland-Altman plots in which the blue dashed lines indicate the mean of the absolute value of the difference (percentage expected minus percentage observed) and the orange dashed lines indicate the SDs (2) for this range. Points along Y,0 indicate schools that demonstrated perfect representativeness of the group. Points above Y,0 indicate schools where the group was overrepresented. Points below Y,0 indicate schools where the group was underrepresented.

Informed consent was obtained for 6325 participants but 518 (8.2%) were excluded from this cross-sectional analysis because the child’s race and/or ethnicity was either missing (n = 152), reported as multiracial in studies before 2009 (n = 211), or identified with the “other” race checkbox (n = 155). In total, 104 schools (N = 5807 children) were included in this analysis. Exclusion criteria with counts are provided in eTable 2 in Supplement 1. Table 2 presents the absolute value of the difference between expected and observed by group. Figure 1 shows the Bland-Altman plots and scatterplots for Asian or Native Hawaiian or other Pacific Islander children and Black children. Figure 2 presents the findings for Hispanic children and White children. In the eFigure in Supplement 1, representativeness results are presented for non-Hispanic multiracial children.

**Table 2.** Percentage-Point Magnitudes and Variation in Representativeness of Each Racial and Ethnic Group^a^

Race and ethnicity	Mean difference between expected and observed (95% CI)
Asian or Native Hawaiian or other Pacific Islander	0.45 (−7.76 to 8.66)
Black	0.12 (−15.73 to 15.96)
Hispanic	0.00 (−17.66 to 17.66)
White	−0.72 (−23.60 to 22.16)
Multiple races, non-Hispanic	0.29 (−5.10 to 5.68)

^a^ Data correspond to the point estimates for the lines on the Bland-Altman plots showing the means of the absolute value of the difference in Figures 1 and 2.

Asian or Native Hawaiian or other Pacific Islander children were overrepresented by 0.45 percentage points (95% CI, −7.76 to 8.66), but most schools had small population shares. The association between percentage expected and percentage observed was significant (Spearman ρ = 0.82; *P* < .001; Figure 1A). The scatterplot’s best-fit line fell below the line of identity as the expected percentage for the Asian or Native Hawaiian or other Pacific Islander group increased, suggesting that these children were more underrepresented when they comprised larger shares of the school population. The Bland-Altman plots indicated little variation in over- and underrepresentation across schools, since the CIs were narrow relative to the other racial and ethnic groups.

Black children were overrepresented by 0.12 percentage points with variation (95% CI, −15.73 to 15.96). The positive correlation between percentage expected and percentage observed for Black children was statistically significant (Spearman ρ = 0.94; *P* < .001; Figure 1C). The clustering of points near 0 and 100 on the scatterplot indicated that many schools had populations that were majority Black students, while others comprised only a small Black student population. The best-fit line on the scatterplot fell above the line of identity at higher values, suggesting that schools where Black children comprised larger shares of the population had a greater magnitude of overrepresentation. Notably and unlike any other predominant race and ethnicity, Black children in this study were never underrepresented in schools where they comprised more than 50% of the target grade population.

Hispanic children also appeared to be proportionally represented with notable variation (0 percentage-point difference [95% CI, −17.66 to 17.66]). The positive correlation between percentage expected and percentage observed among Hispanic children was statistically significant (Spearman ρ = 0.94; *P* < .001; Figure 2). Extreme outliers in both directions on the Bland-Altman plots differentiated Hispanic representativeness from that of other racial and ethnic populations.

White children were underrepresented by 0.72 percentage points (95% CI, −23.60 to 22.16). The positive correlation between percentage expected and percentage observed for White children was statistically significant (Spearman ρ = 0.91; *P* < .001; Figure 2). Although the mean difference between percentage expected and percentage observed was negative, this group appeared the least affected by bias in enrollment because the points above and below 0 were evenly distributed across the x-axis. No immediate patterns emerged between the demographic share of a school and its representation of White children.

## Discussion

In this cross-sectional study, we provided a practical approach for quantifying the representativeness of study populations by adapting the Bland-Altman^17^ method and examining the associations between the percentage of baseline study samples relative to each group’s true population parameters by race and ethnicity. The measurement of site-level representativeness with Bland-Altman plots suggested that the mean difference between percentage expected and percentage observed—information traditionally reported in studies and shown in our analysis with the y-axis line—obscures outliers in over- and underrepresentation. By comparing this y-axis line to the spread of individual points (ie, schools), specific outlier sites were identified easily. Moreover, by examining the location of points along the x-axis of Bland-Altman plots, an association was observed between a racial and ethnic group’s share of the overall target population and their representativeness at the site. Illustrating this approach in our pooled data set underscored the importance of moving beyond reporting representativeness with group means alone. We observed that overall representativeness did not appreciably vary across racial and ethnic groups, but mean values obscured substantial within-group variation. We urge investigators to begin incorporating measures of spread into their research (1) to provide a more complete estimate of representativeness and (2) to pinpoint potential barriers and facilitators to study enrollment by race and ethnicity. Future research replicating our approach could maximize the transparency of racial and ethnic reporting, investigate whether our results in a nonrandom sample of community-based studies hold across other community-based studies in the US, and elucidate the degree to which community-based methods affected our findings.^3^

Our method of quantifying study population representativeness can be applied to a broad range of health research and policy contexts. We assessed the representativeness of enrolled children relative to their respective schools. Additionally, the method discussed supports comparisons at many other levels (eg, country, state, zip code, or census tract) or site-based locales (eg, schools, hospitals, or community centers). Our study focused on racial and ethnic representativeness, but the outcome could encompass a wide range of equity indicators (eg, income, age, nationality). For example, data from national cohort studies could be aggregated by state to examine whether the income of enrolled participants matched the mean income of their state. Future replication studies could also determine whether representativeness varies based on the study outcome or disease type. For instance, by pooling data from cancer clinical trials, the racial representativeness of study populations could be examined based on the cancer type, using the hospital as the unit of analysis. Finally, the graphical component of Bland-Altman analysis can be used to obtain additional layers of information by coding points on the graphs to convey characteristics, such as the year of study enrollment, to detect patterns in representativeness over time.

Our adapted Bland-Altman method also holds promise for the more inclusive reach of federal health, nutrition, and education programs. For instance, the US government estimated that 11 million individuals were eligible to receive Special Supplemental Nutrition Program for Women, Infants, and Children (WIC) benefits in 2019, but only 57% did.^23^ Our proposed method could quantify overall and racial and ethnic differences in WIC participation by state. Each point would indicate the zip code with individual plots for each race and ethnicity. Quantifying representativeness with this approach may provide substantial new knowledge for other federally funded programs, such as Medicare, Medicaid, Head Start, and the Supplemental Nutrition Assistance Program. However, such methodological advancement requires the cooperation of government agencies and relies on accurate and accessible sampling-frame data sets for comparison.

Community-based research methods are associated with more representative enrollment outcomes than traditional clinical and population-based approaches.^3,15,24^ However, even the most rigorous and thoughtfully planned community-based studies, such as those in our sample, may face difficulty overcoming barriers to successfully enrolling individuals disproportionately facing adversity. For example, Asian, Black, and Hispanic children may be exposed to more discrimination at schools than their White peers, which directly impedes their health and may affect study enrollment.^25,26,27^ Our findings suggest that Black children, in particular, may be less likely to enroll in community-based studies when they do not belong to their school’s racial majority. Thus, efforts to include context-specific minoritized children are crucial to address rising health inequities effectively. Relatedly, we observed that the racial and ethnic composition of study settings may also incur beneficial effects. Specifically, Black children appeared more likely to be overrepresented at majority Black schools. Future qualitative research assessing inter- and intragroup social cohesion could examine how school and family racial and ethnic relations affect recruitment outcomes, which investigators should incorporate into individualized recruitment plans.^28^ Finally, each study worked in low-income and resource-limited communities. Identifying the contributing factors associated with site-level underrepresentation across racial and ethnic groups is crucial since children from low-income backgrounds remain underrepresented in the pediatric health literature. Overall, the mechanisms that limit the effectiveness of enrolling representative samples in community-based studies are not well understood but were partially revealed by our finding of school-level differences in representation based on the school majority.

### Limitations

A main limitation of this cross-sectional study was the exclusion of enrolled children from the analysis because their race and ethnicity data could not be compared with the NCES Common Core data sets (ie, race listed as “other” in all years and multiracial respondents prior to 2010). Moreover, the magnitude of representativeness should be interpreted cautiously, particularly since some schools had small numbers of individuals of each race and ethnicity; thus, deviations from representation could indicate differences of just a few children. Race and ethnicity are fluid social constructs. Discrepancies between school and study demographic response options could have affected our findings if parents reported the child’s race and ethnicity differently on each survey.

The included studies were led by investigators with expertise in community-based research. The goal of these community-based studies was to enroll eligible children from schools with certain socioeconomic compositions (Table 1). These community-engaged study practices are known to include historically marginalized populations better than traditional methods.^3,15^ Therefore, our representativeness findings may lack generalizability, but that extent should be confirmed with wider application of our technique. Future research could assess how additional factors, such as study design, community engagement, potential study risks, and indicators of social status (eg, parent education), are associated with racial representativeness in pediatric health studies. Notably, such data must be collected on study surveys to overcome limitations of data availability during similar retrospective assessments.

## Conclusions

In this cross-sectional study, we confronted a critical barrier to health equity: the systematic underrepresentation of Asian, Black, and Hispanic populations in pediatric health research. Our approach for quantifying representativeness in health research is both feasible and replicable. Prevention of the racial and ethnic health disparities that manifest during childhood and persist over the life span requires a paradigm shift during recruitment and when describing study samples and results. Replication and testing of our application of Bland-Altman analysis could help improve the representation of minoritized racial and ethnic populations in the pediatric health evidence base and in health policies and programs. The proposed method may enable investigators to pinpoint the multilevel determinants of enrollment affecting racial and ethnic groups differently, and it could help alleviate preventable chronic disease disparities that are disproportionately endured by US youth from racially minoritized backgrounds.

## Notes

Supplement 1. **eAppendix 1.** Harmonizing Race and Ethnicity Measures Between Study-level and National Center of Education Statistics Data Sets **eAppendix 2.** Comparing the Percent Expected to the Percent Observed to Quantify Representativeness **eTable 1.** Conceptual Terms and Operational Examples to Quantify School-level Racial Representativeness of Study Populations **eTable 2.** Individual Participants Included in Representativeness Comparison and Exclusion Reasons **eFigure.** Variation in the Representativeness of Enrolling Children of 2 or More Races

Supplement 2.
Data Sharing Statement
