# Supplementary material for: Use of Bland-Altman Analysis to Examine the Racial and Ethnic Representativeness of Study Populations in Community-Based Pediatric Health Research
Source: JAMA Netw Open. 2023 May 11;6(5):e2312920. doi: 10.1001/jamanetworkopen.2023.12920 (PMC10176118; doi:10.1001/jamanetworkopen.2023.12920)
Supplement: Supplement 1. — eAppendix 1. Harmonizing Race and Ethnicity Measures Between Study-level and National Center of Education Statistics Data Sets eAppendix 2. Comparing the Percent Expected to the Percent Observed to Quantify Representativeness eTable 1. Conceptual Terms and Operational Examples to Quantify School-level Racial Representativeness of Study Populations eTable 2. Individual Participants Included in Representativeness Comparison and Exclusion Reasons eFigure. Variation in the Representativeness of Enrolling Children of 2 or More Races [file jamanetwopen-e2312920-s001.pdf]

Supplementary Online Content

Krobath DM, Naumova EN, Cuevas AG, Sacheck JM, Wilson NLW, Economos CD. Use of Bland-Altman analysis to examine the racial and ethnic representativeness of study populations in community-based pediatric health research. *JAMA Netw Open*. 2023;6(5):e2312920. doi:10.1001/jamanetworkopen.2023.12920

eAppendix 1. Harmonizing Race and Ethnicity Measures Between Study-level and National Center of Education Statistics Data Sets

eAppendix 2. Comparing the Percent Expected to the Percent Observed to Quantify Representativeness

eTable 1. Conceptual Terms and Operational Examples to Quantify School-level Racial Representativeness of Study Populations

eTable 2. Individual Participants Included in Representativeness Comparison and Exclusion Reasons

eFigure. Variation in the Representativeness of Enrolling Children of 2 or More Races

This supplementary material has been provided by the authors to give readers additional information about their work.

© 2023 Krobath DM et al. *JAMA Network Open*.

eAppendix 1. Harmonizing Race and Ethnicity Measures Between Study-level and National Center of Education Statistics Data Sets

Participant level race and ethnicity data were harmonized according to the National Center of Education Statistics (NCES) reporting categories. Prior to 2010, NCES reported racial and ethnic enrollment with five mutually exclusive categories: 1) Asian, 2) Black/African American, 3) Hispanic, 4) Native American or American Indian, and 5) White. In 2010, NCES updated its reporting procedures. Data are first aggregated by ethnicity and then by race, totaling seven mutually exclusive categories: 1) American Indian or Alaska Native; 2) Asian; 3) Black/African American; 4) Native Hawaiian or Other Pacific Islander (NHPI); 5) White; 6) Two or More Races; and 7) Hispanic or Latino of any race(s). We assessed the representativeness of Asian and Native Hawaiian or Pacific Islanders in a combined category. Participants who were identified on study surveys with the "Other Race" checkbox option were excluded since NCES does not allow reporting children with the category. Multiracial participants recruited prior to 2009 were excluded because NCES did not mandate this reporting category until the 2010-11 school year. American Indian and Alaska Native children were excluded from our analysis due to very low or zero sample sizes. For exclusion criteria and individual counts, refer to eTable 2.

© 2023 Krobath DM et al. *JAMA Network Open*.

eAppendix 2. Comparing the Percent Expected to the Percent Observed to Quantify Representativeness

Each study school was linked to the corresponding National Center of Education Statistics Common of Data using the federal government's unique school-level identifier. For example, to determine the percent expected for a school in a study that recruited only 3rd and 4th graders in 2003, we obtained the 2003-04 Common Core dataset and calculated the total of all 3rd and 4th graders who were reported across the NCES race categories and calculated percent expected and observed as previously described. Therefore, if Hispanic children comprised 60 of the 100 students in grades 3 and 4, we would expect 60% of participants recruited from that school to be Hispanic. But if only 10 of 25 recruited 3rd and 4th graders were Hispanic, we would conclude that this group was underrepresented by 20 percentage points.

© 2023 Krobath DM et al. *JAMA Network Open*.

eTable 1. Conceptual Terms and Operational Examples to Quantify School-level Racial Representativeness of Study Populations

Concept	Operational definition	Example
Representative	The percentage point difference between percent expected and observed was zero.	25 of 50 children recruited at a school were Hispanic/Latino (50% observed) and the population of the target grades at the school was 50% Hispanic/Latino.
Over representative	The percentage point difference between percent expected and observed was positive.	30 of 50 children recruited at a school were Hispanic/Latino (60% observed) and the population of the target grades at the school was 50% Hispanic/Latino. Hispanic/Latino children were **overrepresented** by 10 percentage points.
Under representative	The percentage point difference between percent expected and observed was negative.	20 of 50 children recruited at a school were Hispanic/Latino (40% observed) and the population of the target grades at the school was 50% Hispanic/Latino. Hispanic/Latino children were **underrepresented** by 10 percentage points.

Notes: This table describes how levels of representativeness were defined in the study.

© 2023 Krobath DM et al. *JAMA Network Open*.

eTable 2. Individual Participants Included in Representativeness Comparison and Exclusion Reasons

	**Included in analysis**	**Exclusion Reason**			**Total excluded**
		"Other" Race	*Multiracial (prior to 2009)*	*Race and ethnicity not reported*	
Study A	882	25	NA	76	101
Study B	418	3	NA	27	30
Study C	632	61	NA	0	61
Study D	225	16	NA	0	16
Study E	1245	5	34	18	57
Study F	919	0	25	16	41
Study G	1486	45	152	15	212
Total	5807	155	211	152	518

Notes: Data correspond to individual participants.

© 2023 Krobath DM et al. *JAMA Network Open*.

eFigure. Variation in the Representativeness of Enrolling Children of 2 or More Races

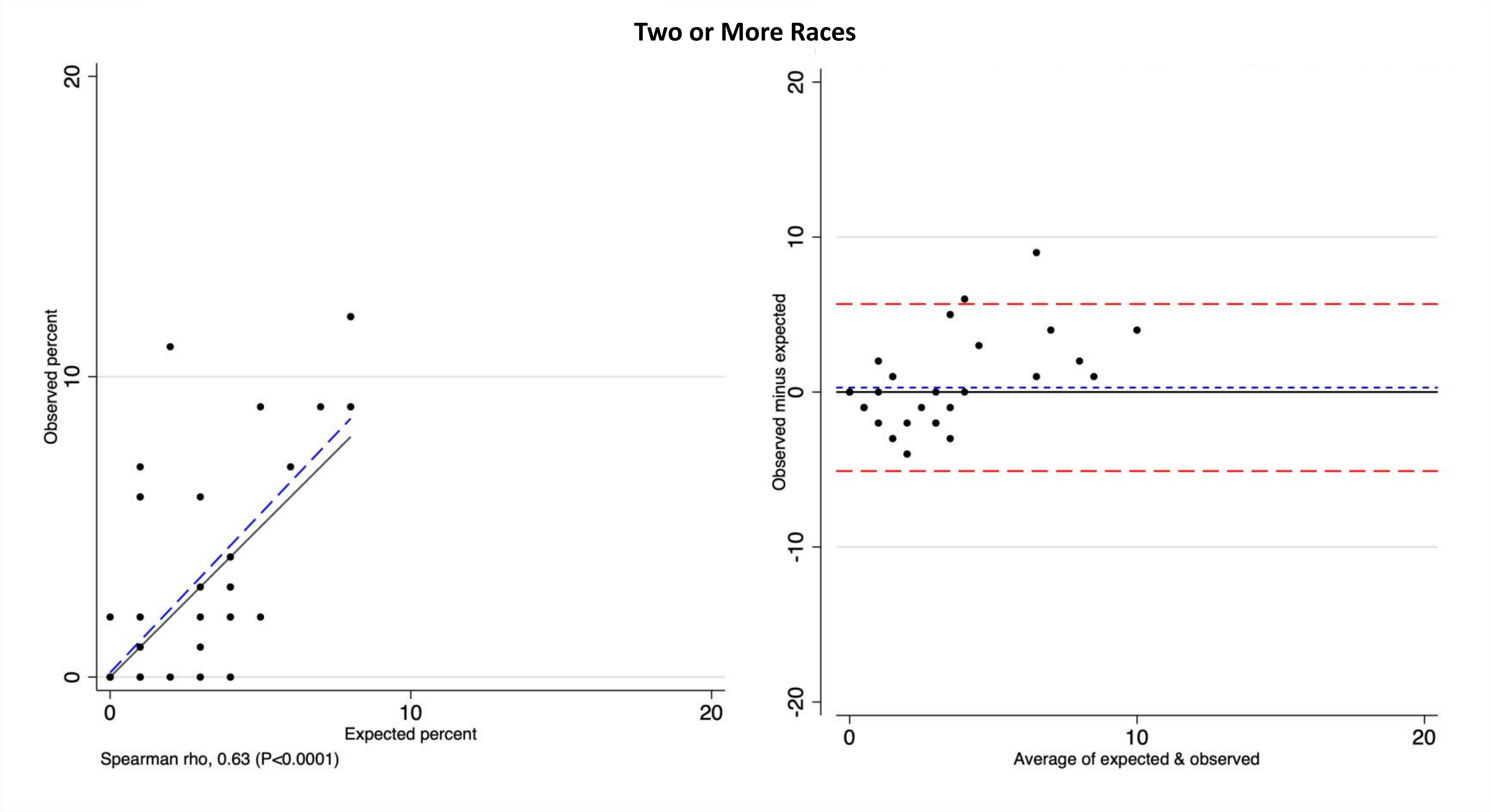

Notes: On the scatterplots (left), the dashed blue line indicates the best fit line, and the solid black line is the line of identity. On the Bland Altman plots (right), the blue line indicates the means of the absolute value of the difference (Expected % minus Observed %) and the red dashed line indicates ±2 SDs for this range. The points along Y, 0 on the Bland Altman plots indicate schools that demonstrated perfect representativeness of the group, the points above Y,0 indicate schools where the group was overrepresented, and the points below Y,0 indicate schools where the group was underrepresented.

© 2023 Krobath DM et al. *JAMA Network Open*.
