# Supplementary material for: Use of Bland-Altman Analysis to Examine the Racial and Ethnic Representativeness of Study Populations in Community-Based Pediatric Health Research
Source: JAMA Netw Open. 2023 May 11;6(5):e2312920. doi: 10.1001/jamanetworkopen.2023.12920 (PMC10176118; doi:10.1001/jamanetworkopen.2023.12920)
Supplement: Supplement 2. — Data Sharing Statement [file jamanetwopen-e2312920-s002.pdf]

Data Sharing Statement

Krobath. Use of Bland-Altman Analysis to Examine the Racial and Ethnic Representativeness of Study Populations in Community-Based Pediatric Health Research. *JAMA Netw Open*. Published May 11, 2023. doi:10.1001/jamanetworkopen.2023.12920

Data
Data available: No

Additional Information
Explanation for why data not available: This is a secondary pooled data analysis and permission to share data was not included in original participant consent forms.
